# Exploring the Reservoir Hosts of Tick-Borne Encephalitis Virus

**DOI:** 10.3390/v11070669

**Published:** 2019-07-22

**Authors:** Anna Michelitsch, Kerstin Wernike, Christine Klaus, Gerhard Dobler, Martin Beer

**Affiliations:** 1 Institute of Diagnostic Virology, Friedrich-Loeffler-Institut, Südufer 10, 17493 Greifswald—Insel Riems, Germany; 2 Institute for Bacterial Infections and Zoonoses, Friedrich-Loeffler-Institut, Naumburger Str. 96a, 07743 Jena, Germany; 3 Bundeswehr Institute of Microbiology, German Center of Infection Research (DZIF) partner site Munich, Neuherbergstraße 11, 80937 München, Germany

**Keywords:** tick-borne encephalitis, ticks, reservoir, transmission, rodent

## Abstract

Tick-borne encephalitis virus (TBEV) is an important arbovirus, which is found across large parts of Eurasia and is considered to be a major health risk for humans. Like any other arbovirus, TBEV relies on complex interactions between vectors, reservoir hosts, and the environment for successful virus circulation. Hard ticks are the vectors for TBEV, transmitting the virus to a variety of animals. The importance of these animals in the lifecycle of TBEV is still up for debate. Large woodland animals seem to have a positive influence on virus circulation by providing a food source for adult ticks; birds are suspected to play a role in virus distribution. Bank voles and yellow-necked mice are often referred to as classical virus reservoirs, but this statement lacks strong evidence supporting their highlighted role. Other small mammals (e.g., insectivores) may also play a crucial role in virus transmission, not to mention the absence of any suspected reservoir host for non-European endemic regions. Theories highlighting the importance of the co-feeding transmission route go as far as naming ticks themselves as the true reservoir for TBEV, and mammalian hosts as a mere bridge for transmission. A deeper insight into the virus reservoir could lead to a better understanding of the development of endemic regions. The spatial distribution of TBEV is constricted to certain areas, forming natural foci that can be restricted to sizes of merely 500 square meters. The limiting factors for their occurrence are largely unknown, but a possible influence of reservoir hosts on the distribution pattern of TBE is discussed. This review aims to give an overview of the multiple factors influencing the TBEV transmission cycle, focusing on the role of virus reservoirs, and highlights the questions that are waiting to be further explored.

## 1. Introduction

The term “arbovirus” describes a group of viruses clustered together solely based on their route of transmission. They have managed to adapt to mammalian hosts as well as to arthropod vectors, adapting their replication cycle to two highly different host organisms. In this regard, it seems even more fascinating that arboviruses are found in more than eight virus families, implementing the emergence of this complex system several times in the course of evolution. Today, there are over 500 arboviruses described, including globally recognized threats to human health such as dengue virus, Zika virus, and Japanese encephalitis virus [1,2].

The main factor for the circulation of any arbovirus is the interplay between the arthropod vector and its (reservoir) hosts. To be maintained within a given region, the virus needs to find a system where there are always sufficient numbers of susceptible hosts for virus amplification and vectors that are able to transmit the virus effectively [3]. Knowing the reservoir of any virus is important to understanding its lifecycle and therefore its distribution. Keeping in mind that this is a complex interplay between many factors, different approaches may sometimes lead to conflicting results. A variety of definitions regarding the term reservoir host are used in the existing literature, with characteristics that often contradict each other [4]. In this review, we present an overview of the current knowledge of the animal hosts involved in the tick-borne encephalitis virus (TBEV) lifecycle and their role in virus maintenance. Without trying to define a classic reservoir host, we aim to highlight the factors contributing to successful virus circulation.

## 2. Tick-Borne Encephalitis: Etiological Agent and Clinical Manifestation

TBEV is one of the main arboviruses in Eurasia, circulating between ticks and vertebrates. It belongs to the family *Flaviviridae*, and within it, to the tick-borne flavivirus group of the genus *Flavivirus* [5]. The genome consists of an approximately 11 kb single-stranded RNA of positive sense, which is packed into an enveloped particle with a diameter of around 50 nm [6]. Based on genome sequence analyses, there are three classic TBEV subtypes described: (I) TBEV-FE (Far East) is found in Asia, mostly in northern China, and in the east of Russia. (II) TBEV-Sib (Siberia) strains are circulating in the rest of Russia, with an outreach to the eastern parts of Europe. (III) TBEV-Eu (Europe) represents the main subtype in mainland Europe [7]. In addition to that, two new subtypes were recently proposed: The Baikalian subtype (TBEV-Bkl), circulating in the region of the Baikal lake [8], and the Himalayan subtype (TBEV-Him), isolated from Himalayan marmots (*Marmota himalayana*) [9]. TBEV evolved in its natural habitat under the constraints of evolution, as part of the specific ecosystem. It adapted to a broad range of species, but remained restricted to natural foci, with strict borders drawn under factors that are still widely unknown to the scientific community [10]. The possible influence of certain weather conditions and adapted host animals is discussed subsequently. TBEV infection leads to the disease tick-borne encephalitis (TBE), also formerly known as Russian spring summer encephalitis (RSSE) in Russia and far eastern Asia, and as Central European encephalitis in the European area [11]. The virus may lead to neurological symptoms varying in severity depending on the subtype. These symptoms may lead to long-lasting sequelae that burden the patient for years after infection, and can also be fatal. Although effective vaccines are available, there are still up to 12,000 cases reported in Europe and Russia each year [12,13]. Disease surveillance in most parts of Asia is not regularly conducted, leaving disease burden estimation to singular outbreak and prevalence studies [14,15]. In addition to human cases, a variety of species are susceptible to TBEV. Rarely, severe clinical symptoms may occur in dogs [16], horses [17], monkeys [18], sheep [19], goats [20], and mouflons [21]. TBEV-specific antibodies have been reported in other animals, such as wild boar, roe deer, or cattle, without clinical disease [22,23].

## 3. TBEV Transmission Cycle: The Tick Vector

For TBEV transmission, the arthropod vectors are primarily hard ticks. In Europe, the most important tick vector is *Ixodes ricinus*, whereas in Russia and Asia it is *Ixodes persulcatus*. In Asia, *Haemaphysalis concinna* also seems to play a major role [24,25]. Other than that, at least 22 tick species have been shown to be able to carry the virus [26,27,28]. Some may be overlooked because of the lack of human infestation, but still contribute to virus circulation, such as *Dermacentor reticulatus* [29,30,31]. This highly adaptive tick species is found in large parts of Europe and Asia, and is often the second most common species. In contrast to the only occasional occurrence of human bites, *Dermacentor reticulatus* ticks surpass the number of *Ixodid* tick bites on large domestic and game animals, leading to a potential additional circulation cycle of TBEV [32,33].

**Figure 1:**
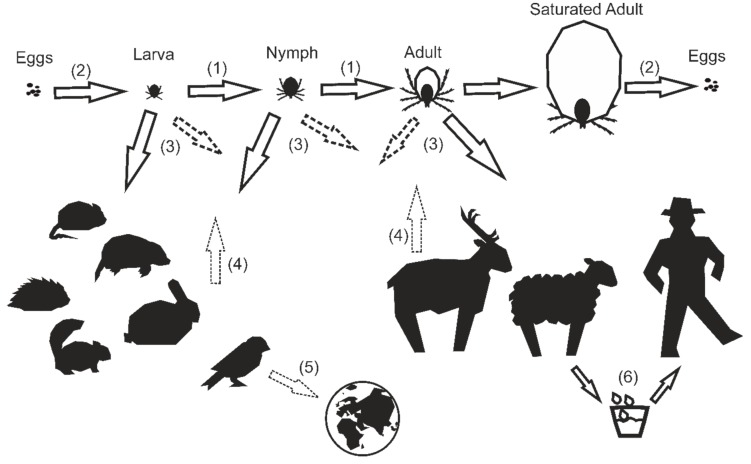
Transmission routes of tick-borne encephalitis virus (TBEV): Infected ticks pass the virus to a variety of small and large animals, as well as humans (**3**). Each stage has a preference for certain animal groups (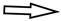), but can be found in a variety of animals (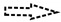). Additionally, humans can become infected by consuming unpasteurized dairy products originating from viremic animals (**6**). Infected birds are suspected to be a vector for virus passage to new endemic foci, although a spatial restriction seems likely (**5**). TBEV is distributed within the tick population mainly through trans-stadial (**1**) transmission, and occasionally through trans-ovarial (**2**) transmission. For successful virus circulation, the virus needs to be spread within the tick population. This is achieved through naïve ticks consuming their blood meal on viremic host animals, as well as through co-feeding (**4**).

The influence of tick population dynamics on TBEV circulation has been reviewed before, highlighting the complex interplay of several factors [34]. In regard to the reservoir function of ticks, two mechanisms play an important role. The virus is maintained in the tick population through trans-ovarial and trans-stadial transmission, meaning that an infected tick can pass the virus through its eggs to its offspring and that the infected tick carries the virus through all life stages, namely the four development stages: eggs, larvae, nymphs, and adults (Figure 1). Through this inner population circulation, TBEV could possibly transit from an infected egg through all stages to the adult tick and to its eggs again [35]. Although the impact of trans-ovarial transmission is still up for debate [36], the trans-stadial transmission of TBEV is believed to be essential for virus survival in nature, although there are some hints that transmission rates between each stage are not as high as expected [37]. Their long lifespans of up to six years and their ability to survive over winter may also help in retaining TBEV for a long period of time in the same places [38,39]. In addition, TBEV influences the behavior of infected ticks, causing an increase in questing activity [40]. All these factors make the vectors themselves a reservoir for TBEV. However, this alone does not seem to be sufficient for virus maintenance. For successful virus circulation, there needs to be an amplifying host reservoir.

## 4. TBEV Transmission Cycle: The Mammalian Reservoir Hosts

**Figure 2:**
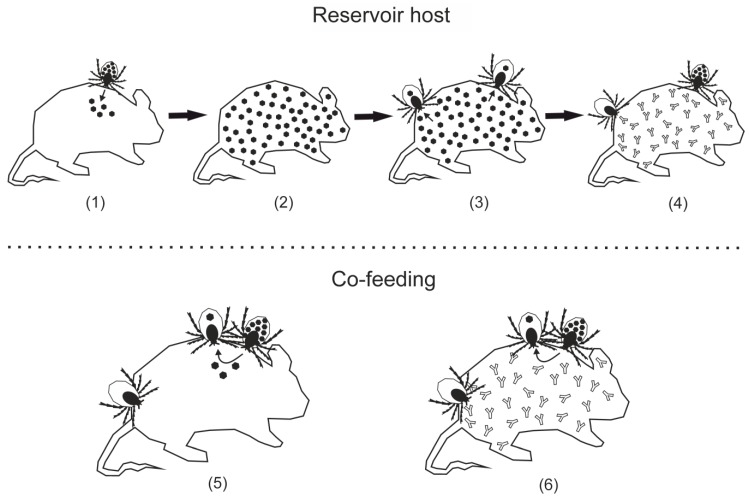
TBEV reservoir hosts: Small mammals, especially rodents, are considered to be reservoir hosts for TBEV. Infected ticks transmit the virus (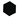) to the animal host (**1**), leading to viremia (**2**). Naïve ticks acquire TBEV by consuming the blood of a viremic host (**3**). As soon as viremia comes to an end, this route of transmission is blocked by circulating antibodies (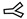) (**4**). Co-feeding enables ticks to pass TBEV among themselves without the need for a viremic host. When naïve ticks feed in close proximity with an infected tick, the animal host acts as a transmission bridge (**5**). This can take place even when the host has antibodies against TBEV (**6**).

For a long time, the consumption of blood from a viremic host by a naïve tick was considered to be the main route of virus dissemination within the tick population. A suitable reservoir host would be an animal that becomes infected with TBEV and keeps the virus circulating in its bloodstream for as long as possible, in titers high enough to infect a feeding tick, without dying from infection, to allow other ticks to feed on it and become infected as well. The effect of co-feeding has also been described, proposing a different method of virus transmission [41]. Through the simultaneous feeding of an infected tick, as well as uninfected ticks in close proximity on the same animal, even when already immunocompetent against TBEV, successful virus transmission is possible without viremia of the host [42] (Figure 2). This mechanism takes advantage of the relatively long phase of feeding on the host, enabling sufficient virus transmission.

Regarding the theory of co-feeding, ticks are considered to be their own reservoir hosts, using the animal to which they are attached as a bridge for transmission. *Ixodes ricinus* and *Ixodes persulcatus* are understood to be the main hosts for TBEV, solely because the larvae and nymphs of these two species tend to have overlapping times of activity, enabling co-feeding between these juvenile stages [43].

Both transmission methods theoretically lead to successful virus amplification and to a spread among the vector population. To which degree the two routes influence the overall virus circulation is still up for debate [44,45].

Human infection can occur through a bite from a TBEV-infected tick when an endemic area is entered. In the period of the year when the tick population reaches its peak, case reports are also at their height, with a delay of three to four weeks. In addition to tick bites, infection through the consumption of unpasteurized dairy products is possible and has now also been reported from Germany [46,47] (Figure 1). In contrast to some mosquito-borne flaviviruses, humans do not play any role in virus transmission, due to low viremia [48] and a lack of sufficient numbers of attached ticks to enable co-feeding. Disease outbreak in humans is theorized to be the result of replication in a not yet adapted host organism [49].

As the main reservoir hosts of TBEV, small mammals such as rodents and insectivores are suspected. In addition to this, an influence of larger game on TBEV prevalence through the influence of mainly adult ticks is discussed (Figure 1). Tick infestation is a major factor in the lifecycle of TBEV, and shows a distinctive pattern for each targeted animal species. There is a general consensus that each tick stage has a certain range of targeted animals, such as adult ticks mainly targeting large animals, while nymphs and larvae stick to small and medium-sized animals, including birds [50,51].

Larger animals, mainly wild cervids, like roe deer (*Capreolus capreolus*) in Europe, are important hosts for adult ticks [52]. They provide a sufficient blood meal for seeking ticks. The population density correlates with the tick prevalence. A higher number of deer leads to a greater tick population, possible influencing TBEV circulation in a positive way [53].

The role of birds in TBEV circulation is not well understood. Multiple bird species, mainly forest passerines, seem to be able to become infected with TBEV; some may even be able to transmit the virus trans-ovarially to their offspring [54]. Tick infestation on birds seems to be related to the amount of time spent on the ground, mainly because of feeding. The ability of birds to easily cross barriers, such as rivers and highways, enables them to spread attached ticks to new areas that animals living on the forest ground might not reach [55]. If the translocated tick finds a suitable environment with the right climatic and fauna conditions, it could distribute the pathogen it carries [56]. The main role of birds is suspected to be in the dispersion of the virus to new endemic regions, as is theorized for many other tick-related pathogens [57]. As discussed in Klaus et al. [51], dispersion over longer distances seems unlikely for TBEV, since *Ixodes* ticks show a relative short feeding period (5 to 9 days) on their avian host in comparison to the amount of time it takes the bird to cover a certain distance. This leads to early detachment, and therefore to a restriction of the distance covered while being attached to the bird.

A perfect virus amplification reservoir is a host that becomes infected easily, maintains the virus for a long time without causing severe symptoms, and provides a constant stream of naïve individuals [58]. In this sense, for an arbovirus transmitted by ticks, small mammals seem to be a good choice. Through their high reproductive rate and relatively short lifespan, there may always be animals that are naïve to the virus and are able to show viremia after a tick bite. Abundant rodent species in European forests, mainly from the Genera *Myodes* and *Apodemus*, show no reproductive limitation to vegetation season, providing young, naïve individuals even in spring, when tick activity is on the rise [59,60].

Small vertebrate animals live close to the ground and are therefore very easy targets for ticks. In rodents, ticks, particularly nymphs and larvae, aggregate in the area behind the animal’s ears, making them an efficient host for transmission through co-feeding. In mainland Europe, the majority of ticks found on trapped rodents originated from only about 20% of captured animals, with two or more nymphs attached to one individual alongside up to 100 larvae. In strong contrast to this, a different picture has been reported from the UK, where only one nymph at most and approximately 10 larvae were found per infested rodent, leading to a nearly 30% increase of the rate of possible co-feeding in European endemic areas in comparison to an area that is naïve to TBEV [61]. In terms of the efficiency of co-feeding, there is a certain dependency on the species it takes place on. The yellow-necked mouse (*Apodemus flavicollis*) seems to be the most adapted species to TBEV and to *Ixodes ricinus* ticks. They show a significantly higher transmission rate than the bank vole (*Myodes glareolus*), which is the second common rodent species in European forests [62]. In addition to this, there are other highly infested small mammals living in tick habitats, such as the two European hedgehog species *Erinaceus roumanicus* [63] and *Erinaceus europaeus* [64], which might provide an equally efficient system.

Studies have provided evidence that TBEV can be passed from experimentally infected voles to their offspring. This vertical transmission allows the virus to circulate within the rodent population without the need for vectors. In the natural reservoir population, this could be a factor that supports long-time virus persistence in a natural endemic focus. However, there are no data available about how passage among only rodent hosts affects the virus and its ability to re-infect arthropod vectors [65]. Experimental infections of suspected reservoir hosts indicate a subclinical infection, with long-lasting virus persistence in the brain. So far, there has been only one study known to us that tried to determine the duration of viremia through PCR analysis of blood samples, indicating a relatively short viremia for the used TBEV-Eu strain, which confirms the results from studies conducted over the last century describing viremia until approximately 4 to 9 days post infection (dpi). In this study, only a single animal showed viremia for up to 84 dpi when infected with the TBEV-Sib strain. In animals that were inoculated with TBEV-FE, TBEV RNA could be detected in the blood for up to 14 dpi [59,66,67,68].

## 5. TBEV Prevalence in Wild Small Vertebrate Hosts

In an attempt to locate possible endemic areas, there have been some prevalence studies on wild animals in certain regions. Besides the testing of wild game and farm animals, rodents have been the main focus of surveillance. For this purpose, wild small vertebrate animals were trapped over a certain amount of time and then examined for TBEV contact either through RT-PCR on organ samples or, in most cases, through the detection of antibodies in blood samples [63,69,70,71,72,73,74,75,76] (Table 1).

In Europe, the two rodent species which lead the studies regarding the number of caught individuals are bank voles and yellow-necked mice [63,69,70,71,72,73,74,75,76]. Besides the yellow-necked mice, two other *Apodemus* species were frequently caught in Europe, the wood mouse (*Apodemus sylvaticus*) [69,70,71,72,73,74,76,77] and the striped field mouse (*Apodemus agrarius*) [69,71,74,75]. Although there were no other *Myodes* species found in these studies, there were some species found from the closely related *Microtus* genus, both *Myodes* and *Microtus* being genera of the subfamily *Arvicolinae*. These species were the common vole (*Microtus arvalis*) [69,72,73,74,76,77] and the European pine vole (*Microtus agrestis)* [69,72,73,74,78], as well as the field vole (*Microtus subterraneus*) [74,75] and the tundra vole (*Microtus oeconomus*) [74,76]. All these species, except for the field and tundra voles, for which the number of caught animals was far too low to draw any conclusions for the whole population, seemed to be in constant contact with TBEV, showing antibodies as well as positive RT-PCR results to various degrees throughout Europe [63,69,70,71,72,73,74,75,76]. In Russia, where the area around Novosibirsk has been the main area of investigation so far, a high prevalence of TBEV was also found in the local *Apodemus* and *Myodes* species, namely the striped field mouse (*Apodemus agrarius*) and the northern red-backed vole (*Myodes rutilus*), as well as the grey red-backed vole (*Myodes rufocans*) [79,80]. Furthermore, the common shrew (*Sorex araneus*) and the Northern birch mouse (*Sicista betulina*) were also found in high numbers, with a high percentage of animals found positive for TBEV RNA. The prevalence of TBEV antibodies, as well as the RT-PCR results, found in rodents caught in those areas was considerably higher than in the European studies [79,80].

The grey red-backed vole was also found TBEV-positive in a Japanese surveillance study of the known TBEV endemic region Hokkaido, as well as the large Japanese field mouse (*Apodemus speciosus*), and the small Japanese field mouse (*Apodemus aregenteus*) [81]. In an additional study, mainly conducted in non-endemic regions of Japan, the most caught species, also being the large and small Japanese field mice, showed no signs of contact with TBEV. Additionally, six other caught small mammal species, mainly the Japanese grass vole (*Microtus montebelli*), were found to be negative for TBEV, noting that no grey red-backed vole could be caught in the non-endemic area of Japan [82].

A small study conducted in South Korea found TBEV in striped field mice, offering no information on overall caught rodents but showing the circulation of TBEV in a country where there has been no notified human case of TBE. The sequenced strain clustered with the TBEV-Eu subtype, which is not to be expected in an Asian country [83]. In addition to that, there have been some known isolations of TBE-Eu in Siberia [84]. A possible explanation would be the entry of an infected tick through migratory birds [57], but, as mentioned above, this theory still lacks data and seems unlikely for TBEV. Another possibility is introduction due to the massive worldwide movement of goods. The importance of these anthropogenic factors in the distribution of TBEV has been shown in a phylogenetic study by Kovalev et al. [85], linking the spread of TBEV-Sib throughout Russia to the construction of the Trans-Siberian Way [3].

In Finland, a study was carried out in two different trapping sites, comparing a TBEV-Sib endemic region with another one endemic for the European subtype. The location, known for the circulation of TBEV-Eu, found field voles as a dominating TBEV-infected species. No bank voles or yellow-necked mice were caught at this site. The TBEV-Sib locus found bank voles, the main species of the other European studies, with TBEV antibodies, as well as the virus in organ samples [78].

Closely related species seem to be able to take over the role as the main reservoir host in the absence of the original host, with field voles taking over for bank voles, and bank voles also being able to circulate TBEV-Sib virus strains [78]. However, due to the small sample size, this might just be the result of local infection pressure, rather than an actual adaption to the respective species. Bank voles were also shown to be susceptible to all three subtypes by experimental infection [66]. Similar to these findings in rodents, tick species seem to be equally susceptible to virus strains of variable subtypes. In studies conducted in Finland, in field-collected *Ixodes persulcatus,* the European subtype was found, and the Siberian subtype was also detected in *Ixodes ricinus* [86,87]. There is a consensus that *Ixodes ricinus* and *Ixodes persulcatus* are the main driving forces for the relatively strict distribution of virus subtypes [27]. Since these studied areas are on the border between the two subtypes, they offer a good place to investigate virus evolution, as well as the interface of the different hosts. Finding one tick positive for an unsuspected subtype may be seen as proof of adaption in different tick species. In addition to this, there is a division inside the *Ixodes persulcatus* population, with two races showing significant variation in morphometric parameters, aligning with the geographic distribution of TBE-Sib and TBE-FE [88].

Although there is no striking connection between host animals and endemic regions, a closer examination of supposedly homogenous mammalian populations could offer an explanation. While there is a concordant geographical distribution of genetic lineages of various animal species around the majority of the world, in Europe, such a pattern cannot be found. In contrast, studies based on mitochondrial DNA analysis reveal distinct distribution patterns of lineages even between mammalian species of the same genus, leading to a high ecological plasticity of many species across Europe [89]. Difference between different lineages, in particular relating to the immune system, might make a species much more diverse than predicted [90].

Next-generation sequencing could be the key to discovering differences within lineages of animal species that might be responsible for different reactions to virus infection, and, as a consequence, potentially influence the development of TBEV endemic areas [91]. A similar situation has already been shown for the distribution of *Puumala orthohantavirus*, since the spatial distribution of this virus is connected to different linages of its reservoir host [92]. Considering bank voles as a potentially important reservoir for TBEV, the relatively closely studied lineage distribution has shown an alliance with TBEV risk areas [93,94].

## 6. Distribution

Over the last few decades, the prevalence of TBEV has been increasing and more endemic regions have been described [95]. This is, on one hand, due to improved surveillance and increased awareness of the possible TBEV infection of most patients suffering from encephalitis. On the other hand, higher temperatures are leading to prolonged tick activity and an increased geographical distribution of ticks, in particular, in northern European countries [96]. Combined with a change in leisure activity, which leads to more frequent visits to tick habitats, this increases the possibility of contact between humans and infected ticks [95].

Multiple factors play a role in the development of a TBEV endemic region. Certain botanical, zoological, climactic, and geo-ecological conditions need to be fulfilled to create a suitable environment for virus circulation [97]. A temperature level of more than 7 °C and a relative humidity of over 80% for most of the time create a suitable tick environment. These conditions are found mainly in forests and grassland areas with sufficient rainfall [96,98]. With regard to TBEV, there are some theories about certain weather conditions promoting the virus circulation [99,100,101]. For example, a rapid fall in ground-level temperatures in early autumn seems to prepone the activity of larvae, adjusting it to the main activity period of nymphs. The resulting enhanced synchronicity of larvae and nymph activity allows a prolonged period of co-feeding between ticks of both stages, and increases the virus transmission rate inside the local tick population [102]. In addition to this, the mere presence of several larvae on the same animal seems to play a major role. Mass co-feeding of larvae in spring as well as in autumn also seems to contribute to virus distribution between ticks to a considerable extent [36].

While the eastern subtypes seem to show quite a homologous distribution alongside the tick population, the European subtype shows a different pattern [102]. Showing lower prevalence in ticks and caught wild rodents, the circulation of TBEV-Eu seems to be restricted by certain factors. Even though ticks and small mammals can be found all around the European continent, TBEV is not endemic in large parts, namely in the west.

Inside an endemic region, TBEV exhibits a specific distribution. In contrast to other tick-borne pathogens like *Borrelia burgdorferi* s.l., TBEV-Eu is not found evenly among the tick population, but is clustered to certain areas from about a few square meters to several square kilometers in size [103,104]. These so-called “natural foci” are believed to be their own autonomous ecosystems, although not showing any striking ecological differences to the surrounding area. There are indications for a center of virus maintenance, in which a constant high infection rate is found in ticks, and which is surrounded by an area where pathogen circulation is significantly lower [105]. Based on studies of two endemic regions in Bavaria, the actual area of a circulating virus strain in this certain area was estimated to be only around 2.500 square meters, with the virus circulating between ticks and small rodents. Out of these so-called “microfoci,” infected ticks may be brought out of the reservoir through medium- and large-sized wild animals. This may lead to transmission in an area of about one kilometer in diameter around the microfocus, described as the “macrofocus” [106]. Existing foci seem to be able to develop in different ways. A study comparing recent data with results from 40 years prior in Thuringia showed that singular foci evolved differently despite being in the same area. Areas with foci of low TBEV incidence showed more human cases of TBEV, while one high-risk focus disappeared completely [107].

## 7. Situation of other Tick-Borne Flaviviruses

When studying the TBEV reservoir, it may also help to take a closer look at its close relatives that induce similar clinical symptoms. Powassan virus (POWV) is a flavivirus from the tick-borne encephalitis serogroup that is mainly found in eastern Russia and North America, including parts of Alaska [108]. Vector ticks are mainly the local *Ixodid* species, like *Ixodes scapularis* and *Ixodes cookei* in America. In Siberia, *Haemaphysalis longicornis* is known to transmit POWV [109]. A serological survey was conducted to get an overview of the POWV prevalence in free-ranging small vertebrate animals. Although there was no further specification about the detected flavivirus, the study gave the first hint of a correlation between small animals and POWV outbreaks. In Siberia and central Alaska, the only species found to be positive for antibodies against the flavivirus serogroup, and by far the most caught species, was the northern red-backed vole (*Myodes rutilus*), which is the same species that dominated Russian survey studies for TBEV [110].

In the south of Alaska, the role of the northern red-backed vole is taken over by the southern red-backed vole (*Myodes gapperi*). In southwestern USA, no *Myodes* species was caught. The most frequently trapped animals were from different species of the genus *Peromyscus*, a genus from the same family as the genus *Myodes.* Some of them were seropositive for some kind of flavivirus that could not be further characterized [110]. In a study conducted in the eastern part of the USA, a *Peromyscus* species, the white-footed mouse (*Peromyscus leucopus*), also made up the largest portion of caught animals. Even though POWV was successfully isolated from ticks from the same area and antibodies against POWV were detected in slightly larger mammals like woodchucks (*Marmota monax*), no antibodies could be found in the white-footed mouse [111].

While POWV shows a similar reservoir situation to TBEV, the tick-borne flavivirus that dominates in Britain, namely the louping ill virus (LIV), has adapted in a different way to the local circumstances. LIV is another tick-borne flavivirus that seems to have only relatively recently diverged from the TBEV complex [112]. The vector tick is *Ixodes ricinus*, the same tick species that transmits TBEV in Europe. However, LIV seems to have adapted to the different environmental conditions of Britain, leaving the woodlands of Europe for the locally more frequent upland moors, switching to sheep (*Ovis aries*) and red grouse (*Lagopus lagopus scoticus*) as the main hosts and resulting in a complete abandonment of small rodents as an important reservoir [113]. LIV also causes actual disease in sheep, switching the main concern to economic losses in agriculture, rather than human infection [114].

## 8. Discussion

TBEV is a tick-borne virus circulating among mainly tick vectors and a variety of vertebrate hosts, and, as in any other biological system, many factors contribute to its lifecycle. Hard ticks play a major role in the distribution of the three virus subtypes across Europe and the northern parts of Asia. Although there is a classic view of small mammals being the major reservoir hosts for virus circulation, there are other important factors that should not be overlooked. Ticks themselves represent a reservoir, circulating the virus within their population mainly through trans-stadial transmission for long time periods. *Myodes glareolus* and *Apodemus flavicollis* make up the majority of mammals caught in European TBEV surveillance studies, and are consistently found positive for antibodies against TBEV, as well as for viral RNA. In Japan, this role is taken up by the local species of the same genera, namely *Apodemus speciosus* and *Myodes rufocans;* in Russia it is *Apodemus agrarius* and *Myodes rutilus*, alongside a high percentage of *Sorex araneus*. Nevertheless, there is a lack of studies on the true potential of these rodents as classic virus reservoirs for TBEV. Most studies, dating back to the middle of the 20th century and mainly written in Russian, go widely unnoticed by the recent scientific community [115]. Since the only recent study concerning viremia of natural rodent hosts hints at a relatively short viremia for TBEV-Eu [66], high prevalence findings within the rodent population of Europe might be a consequence of contact to TBEV-positive ticks. There are only few scientific data about the virus titer of viremia in a potential reservoir host needed for efficient virus transmission [116]. The high titers of TBEV-RNA found in organ samples for a relatively long time after experimental infection could enable active virus transmission through rodents. As shown for other pathogens, tick saliva is able to act as a chemotactic agent, reactivating pathogens and enabling attached ticks to still become infected [42,117].

There is a need for additional experimental infection studies, as well as studies in natural environments, as standardized laboratory conditions might affect the results [118]. Without further proof for the role of ticks as important reservoir hosts, further studies should also focus on a broader range of mammalian hosts. There have been almost no survey studies on other animal species living in tick habitats that might not be trapped in standard devices and do not belong to typical game animals. A study from Kožuch et al. indicates that hedgehogs and dormice (*Glis glis*) seem to be able to carry viruses through hibernation, which might play a role in virus maintenance, but clearly needs further analysis [119]. In regard to co-feeding, there is a lack of further studies after the initial studies made by Labuda [41,62], leading to an acceptance of the mechanism as a side transmission route without exploring its true significance for the overall TBE lifecycle. Furthermore, there are no data available concerning the same effect with the eastern branches of ticks, viruses, and small mammalian animals. Since tick stages seem to meet on a huge variety of animals, co-feeding might be possible on other hosts as well. 

The TBEV lifecycle still offers many unanswered questions ready to be explored, especially if we want to understand its influence on the typical focal distribution of endemic regions. A lot of influence seems to stem from climatic conditions on ticks themselves, as well as on their food sources. Fluctuations of rodent, deer, and tick populations seem to play an unclear role in productive virus transmission. Overall, there is a need for further investigation into the often highlighted role of particular rodent species as a virus reservoir. More in-depth studies of known natural foci and experimental studies on suspected rodent reservoir hosts may provide a better understanding of the complex TBEV lifecycle.

## Figures and Tables

**Table 1:** Small mammalian animals caught in TBEV studies worldwide. Studies focusing on antibody prevalence are shaded in grey; the remaining studies were conducted by screening for viral RNA. CHE—Switzerland; CZE—Czech Republic; DEU—Germany; FIN—Finland; HUN—Hungary; JPN—Japan; KOR—South Korea; RUS—Russia; SVK—Slovakia; SVN—Slovenia.

Genus		Apodemus			Myodes		Microtus					Sorex		Sicista
*Species*		*A. flavicollis*	*A. sylvaticus*	*A. agrarius*	*My. glareolus*		*M. arvalis*	*M. agrestis*	*M. subterraneus*		*M. oeconomus*	*S. araneus*	S. sp.	
		pos./total	pos./total	pos./total	pos./total		pos./total	pos./total	pos./total		pos./total	pos./total	pos./total	
Country	Publication													
CZE	[73]	2/144	0/17		2/92		0/8	0/3						
	[72]	0/77	0/34		1/41		0/2	0/1				0/1		
SVN	[71]	33/820	7/66	4/160	39/272									
SVK	[63]	18/290			2/14								2/12	
	[76]	130/717	36/408		233/1538		14/161				0/2	4/29		
HUN	[74]	4/100	0/11	4/55	6/150		3/48	0/2	0/31		0/8			
	[75]	12/327		8/174	8/39				0/1					
DEU	[69]	10/123	2/7	3/24	21/163		2/21	7/101						
	[77]	14/103	1/19		14/91		1/2							
CHE	[70]	1/77	3/104		8/152									
FIN	[78]				12/80			17/95				0/23		
*Species*		*A. agrarius*			*My. rutilus*	*My. rufocans*						*S. araneus*		*Si. betulina*
		pos./total			pos./total	pos./total						pos./total		pos./total
Country														
KOR	[83]	5/24												
RUS	[79] ^1^	12/34 (16/34)			25/32 (37/45)	18/39						22/30		14/18
		pos.%			pos.%							pos.%		
RUS	[80]	43.3 ± 9			80.0 ± 9.2							69.2 ± 12.8		
		40.6 ± 8.7			61.9 ± 10.8							83.3 ± 6.8		
*Species*		*A. speciosus*	*A. aregenteus*		*My. smithii*	*My. rufocans*	*M. montebelli*	*M. minutus*				*S. unguiculatus*	*S. sp.*	
		pos./total.	pos./total.		pos./total.	pos./total.	pos./total.	pos./total.				pos./total.	pos./total.	
Country														
JPN	[81]	4/24	1/37			14/95						0/6	0/2	
	[82]	2/455	0/36		0/24		0/47	0/1					0/5	

^1^ RT-PCR was performed on brain as well as blood cell samples (shown in parentheses) from the same animals.
